# Severe Hemobilia from Hepatic Artery Pseudoaneurysm

**DOI:** 10.1155/2011/925142

**Published:** 2011-09-06

**Authors:** Fabio Sansonna, Stefano Boati, Raffella Sguinzi, Carmelo Migliorisi, Francesco Pugliese, Raffaele Pugliese

**Affiliations:** ^1^General Surgery and Videolaparoscopy Department, Niguarda Hospital, Piazza Ospedale Maggiore 3, 20162 Milano, Italy; ^2^Service of Interventional Radiology, Niguarda Hospital, Piazza Ospedale Maggiore 3, 20162 Milano, Italy; ^3^Service of Gastroenterology and Digestive Endoscopy, Niguarda Hospital, Piazza Ospedale Maggiore 3, 20162 Milano, Italy

## Abstract

*Background*. Hemobilia is a rare, jeopardizing complication of laparoscopic cholecystectomy coming upon usually within 4 weeks after surgery. The first-line management is angiographic coil embolization of hepatic arteries, which is successful in the majority of bleedings: in a minority of cases, a second embolization or even laparotomy is needed. *Case Presentation*. We describe the case history of a patient in which laparoscopic cholecystectomy was complicated 3 weeks later by massive hemobilia. The cause of haemorrhage was a pseudoaneurysm of a right hepatic artery branching off the superior mesenteric artery; this complication was successfully managed by one-stage angiographic embolization with full recovery of the patient.

## 1. Introduction

Severe hemobilia complicating laparoscopic cholecystectomy (LC) is a rare, unpredictable, and life-threatening vascular complication commonly occurring within 4 weeks from surgery; in the literature, more than sixty cases have been reported by now [1–24]. Preexisting aneurysms [22, 25] and postsurgical pseudoaneurysms of hepatic arteries are the cause of hemobilia in 10% of cases. LC-related iatrogenic pseudoaneurysms of right hepatic artery (RHA) account for around 60% of cases, those of common hepatic artery for around 30% and those of cystic artery for around 10% [6, 15, 21, 23]. Pseudoaneurysms are often close to surgical clips, and may reach 7 cm in size [12, 14, 15, 22, 26]; bile duct leaks may be associated, but clear visualization of the presence of an artero-biliary fistula by imaging radiologic techniques is seldom obtained. In more than 80% of cases, trans-arteriographic embolization (TAE) is the first and definite treatment; in some cases, reembolization is necessary [2, 4, 10, 18], whereas open or laparoscopic surgery ought to be chosen only in case of unsuccessful coil embolization or when embolization is impossible to accomplish [7, 12, 25]. The pathogenesis of this uncommon but sometimes fatal complication [9, 11] still remains unclear. Mechanical or thermal injuries have been considered responsible, but at the moment, precise suggestions to prevent hemobilia after LC are still lacking. We report the clinical history of a woman with uneventful immediate postoperative course of LC who presented with severe hemobilia and anaemia 3 weeks later. 

## 2. Case Presentation

A 55-year-old woman from Eastern Asia had been living in Europe for many years and underwent LC for cholecystitis. Her past medical history included asthma, no previous laparotomy, and abdominal pain for 5 months. Twenty days before LC she was admitted for jaundice to a medical unit, where abdominal percutaneous ultrasound examination showed a thick walled (9 mm) gallbladder with an obstructing gallstone impacted in the infundibulum, without dilation of intra- and extrahepatic bile ducts. The last time she had been to her native country was one year before; biochemical tests demonstrated that the alanine aminotransferase (AST) level was within the normal ranges of 3–45 U/L, the total bilirubin level was 4 mg/dL (nonconjugated bilirubin 3.3 mg/dL), coagulation tests and platelets were normal; the markers of hepatitis B and C were negative, the white blood cells count was normal (8.000/mmc), the eosinophils count was normal, and Entamoeba Histolytica was absent in stool: consequently, no infectious disease was found, and the cause of jaundice remained unexplained. Endoscopic retrograde cholangio pancreatography (ERCP) with endoscopic papillotomy had definitely ruled out obstruction of the biliary tree, while laboratory tests confirmed the persistence of nonconjugated bilirubin values comprised between 3 and 3.5 mg/dL; insofar her jaundice was attributed to Gilbert's disease. Besides, in a fortnight, she became asymptomatic and was discharged. Elective LC was scheduled, but another twenty days later, she complained again of abdominal pain in the upper right quadrant and was admitted to our surgical unit where we decided to perform LC in emergency. Because the walls of gallbladder were thick and cohesive, dissection by monopolar coagulation from liver bed was demanding and took longer time than usually. No intraoperative complication occurred, and after excision of the gallbladder, intraoperative cholangiography was carried out by laparoscopy, confirming the complete patency and normality of the biliary tree and the absence of stones in bile ducts. The early postoperative course of operation was uneventful, and the patient was discharged 5 days after surgery. The histologic examination was consistent with acute inflammation arisen in the context of lithiasic chronic cholecystitis. Oral feeding continued at home, the patient remained asymptomatic for 2 weeks, until she referred a mild epigastric pain irradiated to the right quadrant, but she did not see a doctor; one week later, she experienced sudden hypotension with melena and was admitted to our emergency service. Blood pressure was 100/60 mmHg, pulse rating was 86 beats/minute, haemoglobin level was 8 gr/dL, hematocrit level was 23%, white blood count was 9.700/mmc, alanine aminotransferase (ALT) level was increased to 838 U/L (normal values 3–45), aspartate aminotransferase (AST) level was elevated to 190 U/L (normal values 0–40), alkaline phosphatase level was within the normal and ranges of 35–129 U/L, coagulation tests and platelets were normal, total bilirubin level was 3.5 mg/dL. Digestive endoscopy showed the presence of blood in the upper gastrointestinal tract, without evidence of ulcers or other diseases causing bleedings from stomach or duodenum. Abdominal computed tomography (CT) showed a small haematoma of 3 cm in the gallbladder bed with no hemoperitoneum or any other peritoneal fluid collection and a iatrogenic pseudoaneurysm of RHA beside titanium clips sized 4 mm without arterial blushing. Resuscitation with transfusional support (3 units of packed red blood cells) allowed the patient to reach hemodynamic stability, then she was sent to our surgical ward.

**Figure 1:**
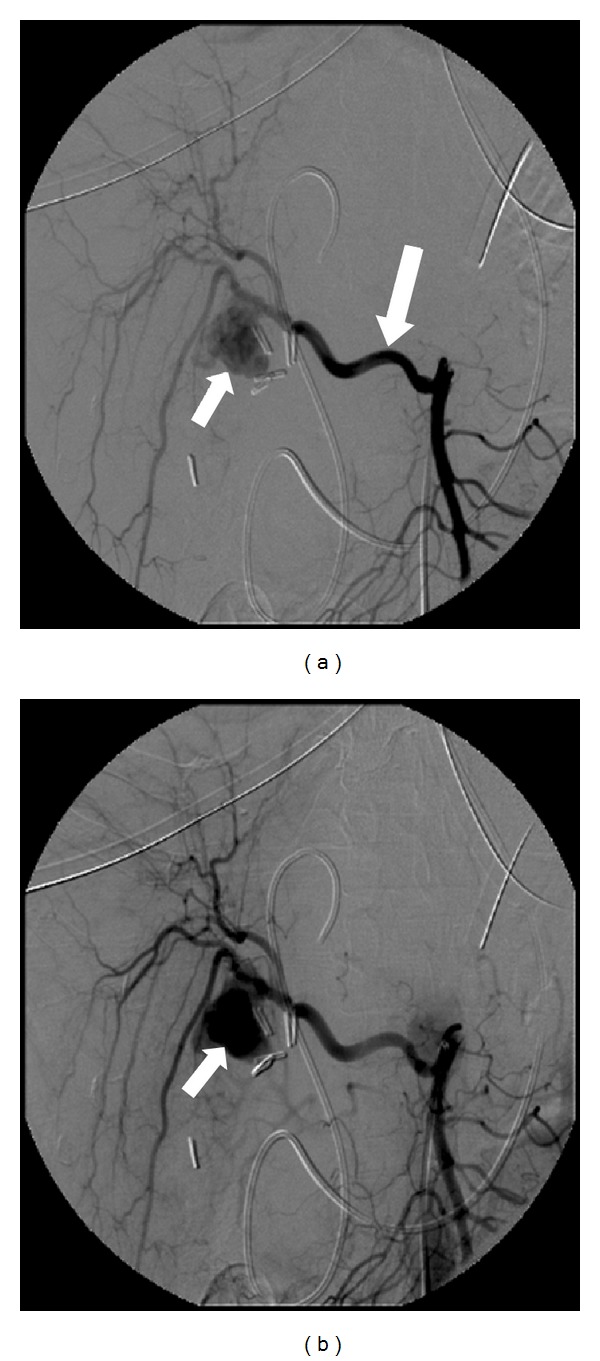
The angiogram shows the sac of a 2 cm pseudoaneurysm, with no radiologic evidence of arterobiliary fistula. A few days before, the CT scan revealed a vascular lesion of 4 mm, successively enlarging to the size reached at the moment of bleeding (smaller arrow). The pseudoaneurysm was located on a replaced right hepatic artery branching off the superior mesenteric artery (greater arrow).

**Figure 2:**
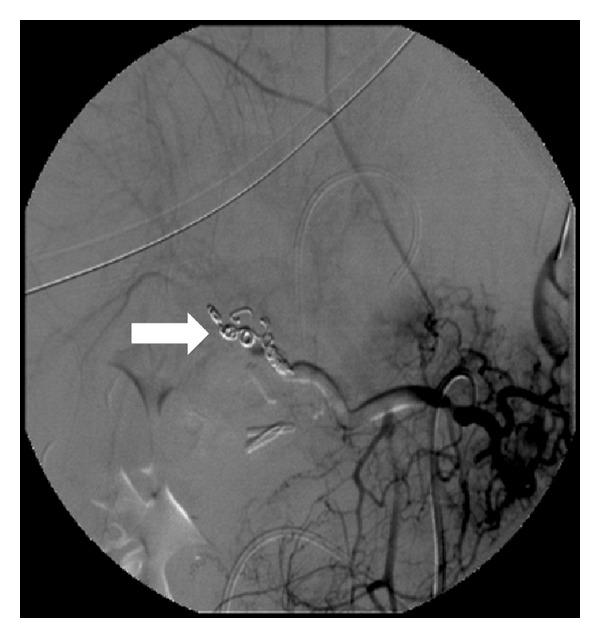
After embolization, the arteriogram shows 3-4 mm coils obstructing the replaced right hepatic artery with complete disappearance of the pseudoaneurysm.

Haemoglobin level increased to 12 gr/dL and hematocrit level to 36%. We excluded surgical primary repair because of the high risks related to possible misinterpretation of anatomy after 3 weeks of local inflammation. Further intermittent episodes of melena occurred in the following days anyway, with hemodynamic stability and minimal decrease of Hb and Ht levels; white blood cells count was 11.000/mmc, alanine aminotransferase (ALT) level lowered to 192 U/L and aspartate aminotransferase to 141 U/L, while the total bilirubin level raised to 12 mg/dL with 2.6 mg/dL of nonconjugated bilirubin. Since the patient persisted stable in hemodynamic parameters without fever or abdominal pain or tenderness, we planned to perform angiography only in case of rebleeding, so much more because CT had not shown any arterial blushing, which could make angiography inconclusive. The patient underwent ERCP that demonstrated a biliary leak in the gallbladder bed at the level of the biliary branch for the V segment; therefore, a nasobiliary drainage (NBD) was placed. Two days later, another episode of severe melena with hemodynamic instability occurred; hence, transfemoral angiography was performed, revealing the presence of a pseudoaneurysm sized 2 cm sited on a replaced RHA with extravasation between the V and VIII segmental branches (Figure 1); RHA was an arterial branch arising from the superior mesenteric artery: TAE was achieved by filling the entire artery and pseudoaneurysm with coils of 3 and 4 mm (Figure 2). The patient had an uneventful clinical course without rebleeding, and the NBD was removed and started oral intake. A CT scan proved revascularization of the right hepatic arterial branches with no ischaemia of the right liver lobe. The patient was discharged 2 weeks later with no impairment of liver function tests, and a magnetic resonance cholangiography showed a normal biliary tree.

## 3. Discussion

The case hereby presented of LC-related hemobilia has been the only one we have registered over the last ten years, accounting for 0.001% of patients with acute cholecystitis operated on in emergency (within 72 hours of admission) and, including elective surgery, accounting for 0.0003% of all the patients undergoing LC over the same span. Hemobilia complicating LC has become a well-known serious event reported in plenty of issues. Symptoms and signs appear within 4 weeks from LC in 80% of cases [19], and only in 3 issues, this complication occurred one year after surgery or even later [12, 19, 21]. Upper gastrointestinal bleeding with melena is the commonest sign of hemobilia and is observed in 90% of cases, whereas abdominal pain is present in 70% and jaundice in 60% of patients; the classic Quincke's triad comprehending melena, pain in the right upper quadrant, and jaundice is observed in 20–40% of patients. In the case described a nonobstructive jaundice was present even before LC; therefore, this sign could not be helpful for diagnostic suspicion. In around 60% of cases, a pseudoaneurysm of RHA is found, in some cases branching off the superior mesenteric artery [12], less frequently false aneurysms of common hepatic artery or cystic artery are found [6, 15, 21]. In the present case, a small pseudoaneurysm of RHA arising from the superior mesenteric artery was the cause of hemobilia, ERCP showed a biliary leak in the hepatic bed, but the existence of an arterobiliary fistula remained unvisualized by the imaging techniques. Allegedly, the incidence of vascular injuries during LC ranges between 0.25% and 0.8% [18, 19], whereas the incidence of biliary injuries ranges between 0.2% and 1% [18, 20]; LC-related hemobilia due to pseudoaneurysm accounts for 4.5% of biliary lesions, that is, around 0.0004% of LC procedures [20], nearly the same as in our experience. TAE of hepatic branches is the first line procedure, whereas open or laparoscopic surgery should be advocated only in case of unsuccessful coil embolization. TAE may be followed by rebleeding and require a second embolization or emergency laparotomy [2, 4, 7, 10, 12]. In the case presented, one single coil embolization of RHA obtained the definite management of haemorrhage. To date, no definite pathogenetic explanation of hemobilia following LC has been given. Because titanium clips are often found next to pseudoaneurysms and monopolar coagulation is usually adopted by laparoscopic surgeons, mechanical and thermal injuries both to biliary and vascular structures have been considered responsible for this complication. If an inadvertent thermal damage occurs, a char of a biliary duct may ensue, followed weeks later by its detachment; bile erosion of a vascular char may also play a role in the pathogenesis of bleeding, while fistulization into the biliary tree may explain hemobilia. Hemobilia may also occur after elective hepatobiliary surgery and emergency open or converted cholecystectomy, during which clips are never (or seldom) employed: instead, severe local inflammation may entail difficult dissection and thermal damage, which must be the real causes of inadvertent vascular injuries in such cases [27–31]. Pseudoaneurysms of hepatic or cystic artery can be even secondary to acute or chronic cholecystitis [26, 32, 33], and perhaps in some cases, this vascular lesion is present even before LC. The size of pseudoaneurysms increases with the time and may reach the noticeable size of 7 cm, as observed when cholecystitis is managed nonoperatively for long time [22, 26] or, less frequently, when the vascular lesion complicates LC and becomes symptomatic long time later [19]. In the case presented, the patient had complained for months of abdominal pain, and the histologic examination showed a thick walled gallbladder with acute inflammation and chronic cholecystitis: the pseudoaneurysm was a tiny one (4 mm), hence inadvertent thermal damage must have been the only real cause of the vascular complication herein described. The cases reported in the literature often refer to surgical histories of difficult, time-consuming LC carrying the risk of inadvertent vascular injuries with occurrence of a pseudoaneurysm thereafter. Suggestions about prevention of such events cannot be found in the specific literature of this complication. Anyway, we have enough data to argue that when dealing with thick walled gallbladders, the adoption of bipolar coagulation or ultrasonic dissection represents a good piece of advice, especially when dissection digs deep into the liver bed. Under such circumstances, possible thermal damages may be prevented by employing ultrasonic coagulation, since the potential carbonization to surrounding tissues is minimal compared to laser [1], monopolar, and even bipolar coagulation [34–37]. Hence, the consequences of inadvertent injuries to biliary structures should be minimized by using ultrasonic instrumentation, and haemostasis in the hepatic bed should be achieved by absorbable hemostats products rather than by monopolar coagulation. When the cystic artery arises low in Calot's triangle, below the cystic duct, the surgeon can suspect the presence of a replaced or aberrant RHA branching off the superior mesenteric artery, which can be found in 5–25% of subjects.

A *replaced RHA* is an artery supplying the right hepatic lobe, whereas an *aberrant RHA* is an additional branch of RHA [38]; if the suspicion of such anatomical variations is present, the surgeon ought to be particularly cautious with coagulation in that area, and ultrasonic dissection should be preferred. Some authors speculate that laparoscopic titanium clips are often found near the pseudoaneurysms and may be partially responsible for arterial or biliary injuries [12, 14, 15, 19, 22]. Determining if thermal damage is transmitted or not through the clips is impossible. Since dissection in Calot's triangle is commonly carried out before firing clips and dissection in the gallbladder bed requires no clip application, transmission of thermal energy by clips is unlikely, too. In order to avoid clip application, if surgeons share this opinion, the cystic artery may be coagulated by bipolar forceps, and the cystic duct may be ligated with absorbable thread using two graspers to tie knots around it. Obviously, there is no evidence that this strategy is effective in preventing chars of arterial or biliary structures: adopting these strategies or not is quite up to each surgeon's choice.

## 4. Conclusion

The occurrence of severe hemobilia following LC is a life-threatening, unpredictable vascular complication that can be managed successfully by TAE. Nevertheless, a means to prevent this vascular complication has not been found yet. Within the purpose of minimizing lateral thermal damage, we propose the avoidance of titanium clips, the avoidance of monopolar coagulation, and the use of absorbable hemostats in the hepatic bed, together with the adoption of ultrasonic devices during difficult dissections.
